# Effects of the commensal microbiota on spleen and mesenteric lymph node immune function: investigation in a germ-free piglet model

**DOI:** 10.3389/fmicb.2024.1398631

**Published:** 2024-06-12

**Authors:** Yan Liu, Jinwei Zhang, Guitao Yang, Chuang Tang, Xiaokai Li, Lu Lu, Keren Long, Jing Sun, Yuchun Ding, Xuewei Li, Mingzhou Li, Liangpeng Ge, Jideng Ma

**Affiliations:** ^1^College of Animal Science and Technology, Sichuan Agricultural University, Chengdu, China; ^2^Chongqing Academy of Animal Sciences, Chongqing, China; ^3^National Center of Technology Innovation for Pigs, Chongqing, China; ^4^Ministry of Agriculture Key Laboratory of Pig Industry Sciences, Chongqing Key Laboratory of Pig Industry Sciences, Chongqing, China

**Keywords:** commensal microbiota, immunity, germ-free piglet, spleen, mesenteric lymph node

## Abstract

Commensal microbial–host interaction is crucial for host metabolism, growth, development, and immunity. However, research on microbial–host immunity in large animal models has been limited. This study was conducted to investigate the effects of the commensal microbiota on immune function in two model groups: germ-free (GF) and specific-pathogen-free (SPF) piglets. The weight and organ index of the spleen of the GF piglet were larger than those in the SPF piglet (*P* < 0.05). The histological structure of the red pulp area and mean area of germinal centers were larger in the SPF piglet than in the GF piglet (*P* < 0.05), whereas the areas of staining of B cells and T cells in the spleen and mesenteric lymph nodes (MLNs) were lower in the GF piglet (*P* < 0.05). We identified immune-related genes in the spleen and MLNs using RNA sequencing, and used real-time quantitative PCR to analyze the expression of core genes identified in gene set enrichment analysis. The expression levels of genes in the transforming growth factor-β/SMAD3 signaling pathway, Toll-like receptor 2/MyD88/nuclear factor-κB signaling pathway, and pro-inflammatory factor genes *IL-6* and *TNF-α* in the spleen and MLNs were higher in the SPF piglet and in splenic lymphocytes compared with those in the GF and control group, respectively, under treatment with acetic acid, propionic acid, butyric acid, lipopolysaccharide (LPS), or concanavalin A (ConA). The abundances of plasma cells, CD8^++^ T cells, follicular helper T cells, and resting natural killer cells in the spleen and MLNs were significantly greater in the SPF piglet than in the GF piglet (*P* < 0.05). In conclusion, the commensal microbiota influenced the immune tissue structure, abundances of immune cells, and expression of immune-related pathways, indicating the importance of the commensal microbiota for spleen and MLNs development and function. In our study, GF piglet was used as the research model, eliminating the interference of microbiota in the experiment, and providing a suitable and efficient large animal research model for exploring the mechanism of “microbial-host” interactions.

## 1 Introduction

The commensal microbiota colonizes various tissues of the body, including the gastrointestinal tract, oral cavity, respiratory tract, urogenital tract, and skin, providing immunomodulatory signals during the development, differentiation, and activation of immune cells, which are important for the maintenance of immune homeostasis (Lloyd and Marsland, 2017; Ganal-Vonarburg and Duerr, 2020). Hosts have coevolved with their commensal microbiota in a complex mechanism that affects innate and adaptive immunity (Alexander et al., 2014; Ganal-Vonarburg and Duerr, 2020). Adaptive immunity mainly comprises B cell-mediated humoral immunity and T cell-mediated cellular immunity (McCoy et al., 2017). The commensal microbiota directly regulates the differentiation and activation of B and T cells (Geuking and Burkhard, 2020), affecting the secretion of immunoglobulin (Ig)A, IgG, IgM, IgD, and IgE by plasma cells, forming the first barrier, and protecting the body from harm via infection (Kim and Kim, 2017). As the first line of host defense, the innate immune system recognizes microbial pathogens with host germline-encoded pattern recognition receptors (PRRs) that bind unique pathogen-associated molecular patterns (PAMPs) (Takeuchi and Akira, 2010).

The spleen, as the largest secondary lymphoid tissue in the body of animals, is divided into different compartments by B and T lymphocytes: follicles containing B cells and periarteriolar lymph sheaths containing T cells (Steiniger et al., 2011; Enders et al., 2020). The most developed lymphoid regions in mesenteric lymph nodes (MLNs) comprise three main areas: follicular region, paracortex, and cortex (Zhang et al., 2012). Dendritic cells (DCs) in the spleen can reach various tissues of the body through the bloodstream, taking up antigens in tissues. After activation of Toll-like receptors (TLRs), cells involved in immune functions present antigens through white pulp to trigger initial activation of T cells, which can induce immune tolerance (Zarember and Godowski, 2002; Yi et al., 2015). Microbiome-derived TLR ligands and metabolites act directly on enterocytes and intestinal immune cells, but can also travel through the systemic circulation to modulate immunity in remote tissues, such as the spleen (Gommerman et al., 2014; Shao et al., 2016). Tight junctions between the intestinal epithelial cells and mucus produced by goblet cells and hematopoietic cells, such as mononuclear cells and innate lymphoid cells (ILCs), including natural killer (NK) cells, are important effector cells of the innate immune system (Hooper and Macpherson, 2010; Eberl et al., 2015). NK cells express T-bet and produce tumor necrosis factor (TNF)-α (Ganal-Vonarburg and Duerr, 2020). The commensal microbiota has been identified as an important regulator of immune cell activation and inflammation, acting through nuclear factor kappa B (NF-κB) and inflammasome activation (Xing et al., 2021). As a key enforcer of immune tolerance and a suppressor of inflammation, transforming growth factor-β (TGF-β) restricts multiple functions of the innate and adaptive immune systems, with Smad3 as an intracellular signaling protein in the TGF-β pathway (Lees et al., 2011; Massagué and Sheppard, 2023). TGF-β not only enhances the B cell response (Reboldi et al., 2016) but also suppresses T cell proliferation and activation through differentiation of regulatory T cells (Tregs) (Li et al., 2006). Furthermore, specific intestinal microbes, possibly acting through metabolites such as short-chain fatty acids (SCFAs), contribute to immune homeostasis by modulating TGF-β production (Pang et al., 2018).

Germ-free (GF) mice have been used to study the effects of the commensal microbiota on the host immune system (Clarke and Bradfute, 2020; Garcia-Flores et al., 2020; Li et al., 2022). However, rodent models are limited by a number of important physiological and metabolic differences from humans (Bayer et al., 2019). In addition, the results of our recent experiments in Peyer’s patches (PPs) of GF piglets (Zhang et al., 2023) are not entirely consistent with those in PPs of GF mice (Li et al., 2023), suggesting that it may be difficult to replicate immune-related research conclusions based on rodent models in large animal models. GF and specific-pathogen-free (SPF) piglets are ideal large animal models for researching microbial–host interactions (Zhou et al., 2021). In this study, we explored the effects of the commensal microbiota on histological structure, gene expression, immune cell composition, and immune-related signal pathways in the host spleen and MLNs in GF and SPF piglet models. Our findings further elucidate the influence of the commensal microbiota on the development of the piglet immune system and support the use of the piglet immunological model as an alternative to widely used rodent models.

## 2 Materials and methods

### 2.1 GF and SPF piglet preparation

All animals were fed at the experimental base of Chongqing Academy of Animal Sciences. The preparation of Bama strain GF piglets and GF environment testing were based on Zhang et al. (2023). The GF piglets were reared in sterile isolators under GF conditions and were handfed Co60 γ-irradiated sterile 4.8%-fat cow milk powder diluted with sterile water (Anyou Group, Jiangsu, China). Once per week, the GF environments were checked for aerobic and anaerobic bacterial contamination (Sun et al., 2018). Of six GF piglets, three were assigned to the GF piglet; the other three were raised in rearing isolators in a barrier environment which the cleanliness could reach 10,000 and required independent flow of clean air with a high ventilation frequency (20 ∼ 60 times/h) (Ogden et al., 2016) and assigned to the SPF piglet. The pathogen detection report of SPF piglet was attached Supplementary Figure 1. The Ethics Committee of the Chongqing Academy of Animal Science reviewed the relevant ethical issues and approved the study (permit number XKY-No. 20210606).

### 2.2 Sample collection

At 42 days of age, all GF and SPF piglets were weighed, euthanized under isoflurane anesthesia, and exsanguinated in a sterile environment, followed by careful removal and weighing of the spleens. The mass of the MLNs in the jejunum was too slight to be weighed. A portion of the fresh spleen and MLNs were fixed with 4% (w/v) paraformaldehyde (Beyotime, China) for histomorphologic analysis. The remainder of the spleen and MLNs samples were snap-frozen in liquid nitrogen, and then stored at −80°C for further analysis.

### 2.3 Hematoxylin-eosin and immunohistochemistry staining

For hematoxylin-eosin (HE) staining, fixed tissues were dehydrated and embedded in paraffin. Tissue blocks were sliced, and the tissue sections were deparaffinized and stained with HE (Beyotime, China). B- and T-cell distributions were examined by immunohistochemistry (IHC) staining using CD3^+^ and CD20^+^ pig-specific makers, respectively. IHC staining was performed following the method described by Zhang et al. (2023). Semi-quantitative evaluation of IHC staining using an Image-Pro Plus (version 6.0) followed the method introduced in a previous report (Wang et al., 2009). Briefly, after B cell and T cell zones were identified based on the structure of the splenic central arteriole and the packing density of the cell populations (van Krieken et al., 1985), parameters including area and integrated optical density (IOD) were measured. The mean optical staining densities of the B-cell and T-cell zones were calculated as: sum of IOD/sum of area.

### 2.4 Total RNA extraction, library preparation, and sequencing

Total RNA was isolated from the frozen spleen and MLNs using TRIzol reagent (Takara Bio, Japan), in accordance with the manufacturer’s instructions. Total RNA samples with a ratio of absorbance at 260/280 nm ranging from 1.8 to 2.0 and an RNA integrity number >8 were used for library construction. We pooled the samples from each tissue by group and prepared a total of 12 sequencing libraries: three replicates each of GF versus SPF in spleen and MLNs. All libraries were constructed and sequenced on the DNBSEQ-T7 seq platform (Novogene, China), and 150-base pair paired-end reads were obtained. High-quality data (clean data) were generated from raw data through rigorous quality control, removing poly-N and low-quality reads, including those with ≥10% N, >10 nucleotides aligned to the adapter with ≤10% mismatches allowed, and >50% of bases with Phred quality <5. The RNA-seq data have been deposited in the GSA with the accession number CRA015219.

### 2.5 Expression analysis of mRNA

Levels of mRNA were quantified using Kallisto software (v 0.44.0), and the transcripts per kilobase of exon model per million mapped reads (TPM) value of each sample was calculated. An mRNA with a TPM value >1 and at least three repeats within one group was considered expressed. Differentially expressed (DE) genes were identified by edgeR (v 3.10) using cutoffs of *P* < 0.05 and | log_2_(Fold Change) | > 1(Bergen and Wu, 2009). Gene functional enrichment analysis and gene set enrichment analysis (GSEA) were performed at the Metascape^1^ (Zhou et al., 2019) and Omicshare^2^ websites, respectively. We uploaded each sample matrix to the CIBERSORTx database,^3^ to calculate the abundances of each immune cell type (Yao et al., 2023), selecting samples with a *P* value < 0.05.

### 2.6 Splenic lymphocyte isolation and culture

Spleens from 42-day-old piglets were aseptically removed and bubbled in pre-cooled phosphate-buffered saline (PBS) (Boster Bio, China) containing 5% penicillin/streptomycin for 3–5 min, followed by removal of the connective tissues around the spleen. After three washes in pre-cooled PBS, the spleen was cut into small pieces. Steps for isolating spleen lymphocytes using the Porcine Spleen Lymphocyte Isolation Kit (TBD Sciences, China) followed the protocol described by Ren et al. (2020). Splenic lymphocytes were suspended in RPMI-1640 complete medium and counted using trypan blue staining exclusion. Experiments were conducted on splenic lymphocytes reaching a concentration of 3.75 × 10^6^ cells/ml with a viability >95%. Splenic lymphocytes were cultured in RPMI-1640 medium (Procell Life Science & Technology Co., Ltd., China) with 10% fetal bovine serum (Procell Life Science & Technology Co., Ltd., China) and 1% penicillin/streptomycin in a 37°C incubator with 5% CO_2_. The cells were divided into five treatment groups: control (treated with Phosphate Buffered Saline) (Servicebio, China), SCFAs (acetic acid, propionic acid, and butyric acid), lipopolysaccharide (LPS) (Beyotime, China), and concanavalin A (ConA) (Beyotime, China). SCFAs concentrations were based on Zhang et al. (2023), LPS and ConA concentrations were based on Cho et al. (2023).

### 2.7 Cell proliferation assays

Cell proliferation was measured using a Cell Counting Kit (CCK)-8 Assay Kit (Beyotime, China) following the manufacturer’s instructions. Splenic lymphocytes were seeded in a 96-well plate (2 × 10^5^ cells/well), divided into three replicates for each of the six treatment groups. After applying each treatment, cells were cultured and assayed at 1, 3, 6, and 12 h by the addition of 10 μl/well CCK-8. Optical density (OD) was measured at 450 nm using a microplate reader (Thermo Fisher Scientific, Spain).

### 2.8 Real-time quantitative PCR

Total RNA was extracted from frozen spleen, MLNs samples, and splenic lymphocytes using HiPure Total RNA Mini Kits (Magen, China), in accordance with the manufacturer’s instructions. Synthesis of cDNA was carried out as described by Pistol et al. (2015), using a PrimeScript RT reagent Kit with gDNA Eraser (Takara Bio, Japan) following the manufacturer’s instructions. Primers were designed through the National Center for Biotechnology Information database and synthesized commercially by Tsingke (China). Real-time quantitative PCR (RT-qPCR) was performed on an ABI Prism 7000 detection system in a two-step protocol with SYBR Green (Applied Bio-Systems, Foster City, CA, USA). Each reaction was performed in a volume containing 1 μl cDNA, 5 ml SYBR Premix Ex Taq (2×), 0.2 μl ROX reference dye (50×), 0.4 μl of each forward and reverse primer, and 3 μl PCR-grade water. The following PCR reaction was performed in a Quant Studio 6 Flex (Thermo Fisher Scientific, USA): 95°C for 30 s, followed by 40 cycles of 95°C for 5 s and 60°C for 34 s, and ending with a melting curve analysis (65–95°C). The relative expression of mRNA was calculated using the 2^–Δ^ ^Δ^ *^CT^* method and expressed as fold-change relative to the GF or cell culture control group. *GADPH* was used to normalize the gene expression. The specific primer sequences in this study are listed in Supplementary Table 1.

### 2.9 Statistical analysis

All statistical analyses were performed using Graphpad Prism 9.5. Unless otherwise stated, numerical data are presented as the mean ± standard deviation (SD). For single comparisons between two groups, the unpaired *t*-test was used. *P-*values < 0.05 and <0.01 were considered to indicate statistical significance.

## 3 Results

### 3.1 Effects of the commensal microbiota on histological structure in the piglet spleen and MLNs

**FIGURE 1:**
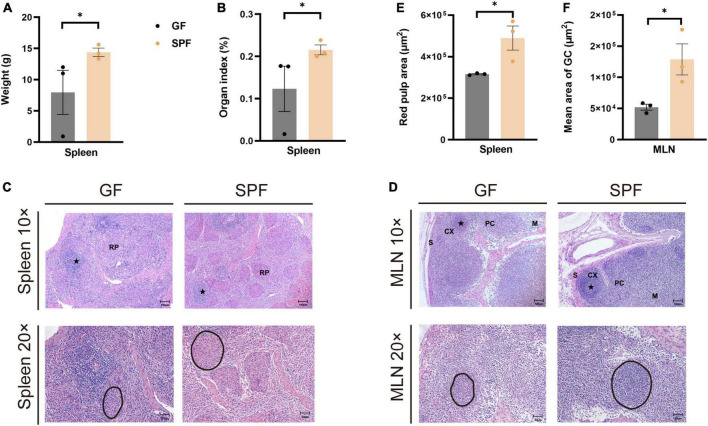
Differences in spleen weight, organ index, and HE staining in GF and SPF piglets. Spleen weight **(A)** and organ index **(B)** in the GF and SPF piglets. **(C)** HE staining of the spleen and quantification of the red pulp area **(E)** in the GF and SPF piglets. **(D,F)** HE staining of MLNs and the mean area of the follicular region **(F)** in the GF and SPF piglets. S, sinus; CX, cortex; PC, paracortex; M, medulla; RP, red pulp. Black circles in panels **(C,D)** indicate red pulp areas and germinal centers, respectively. Data expressed as means ± SD for each group. *n* = 3. *P < 0.05. Scale bars, 50 and 100 μm.

The spleen and MLNs play important roles in innate and adaptive immunity. We established GF and SPF piglets to explore the histological differences in spleen and MLNs between the two models. The spleen weight and organ index were significantly lower in the GF piglet than in the SPF piglet (*P* < 0.05; Figures 1A, B). Although red pulp structures and lymphatic follicles in the spleen were obvious in both piglets (Figure 1C), the red pulp area was larger in the SPF piglet (Figure 1E). The mature lymphoid regions in MLNs were as follows: the follicular region, in which many germinal centers were located; the paracortex, a lymphatic region located between the cortex and the medulla; and the sinusoid region, comprising the lymphatic cord and sinus (Figure 1D). The mean area of the germinal center was larger in the SPF piglet than in the GF piglet (Figure 1F). These results indicated that the commensal microbiota promotes the development and maturation of the spleen and MLNs in piglets.

### 3.2 Effects of the commensal microbiota on the distribution of B cells and T cells in the piglet spleen and MLNs

**FIGURE 2:**
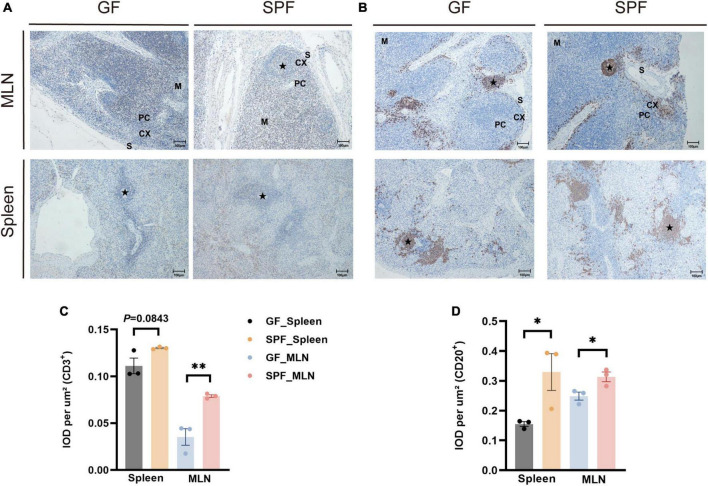
Distribution of B cells and T cells in the spleen and MLNs. IHC staining of B cells and T cells using pig-specific CD3^+^ **(A)** and CD20^+^ **(B)** antibodies. Scale bar, 100 μm. **(C,D)** Quantitative analysis of the proportions of CD3^+^ and CD20^+^ cells. Data expressed as means ± SD for each group. *n* = 3. *P < 0.05, **P < 0.01. IOD values may reflect the comprehensive changes in OD and area of the measured structure.

The spleen and MLNs contain a variety of immune cells that co-participate in B cell-mediated humoral immunity and T cell-mediated cellular immunity (McCoy et al., 2017). IHC staining of porcine-specific B and T cell markers revealed that B and T cells were mainly distributed in the lymphatic follicles of the spleen and in the cortical part of the MLNs (Figures 2A, B). The proportions of T cells were slightly higher in the spleen of the SPF piglet than in the GF piglet (*P* = 0.0843) and were significantly higher in the MLNs of the SPF piglet than in those of the GF piglet (*P* < 0.05; Figure 2C). Furthermore, the proportions of B cells were significantly higher in the spleen and MLNs of the SPF piglet than in those of the GF piglet (*P* < 0.05; Figure 2D), indicating that B and T cell development in the spleen and MLNs are influenced by colonization of the commensal microbiota.

### 3.3 Effects of the commensal microbiota on mRNA expression in the piglet spleen and MLNs

**FIGURE 3:**
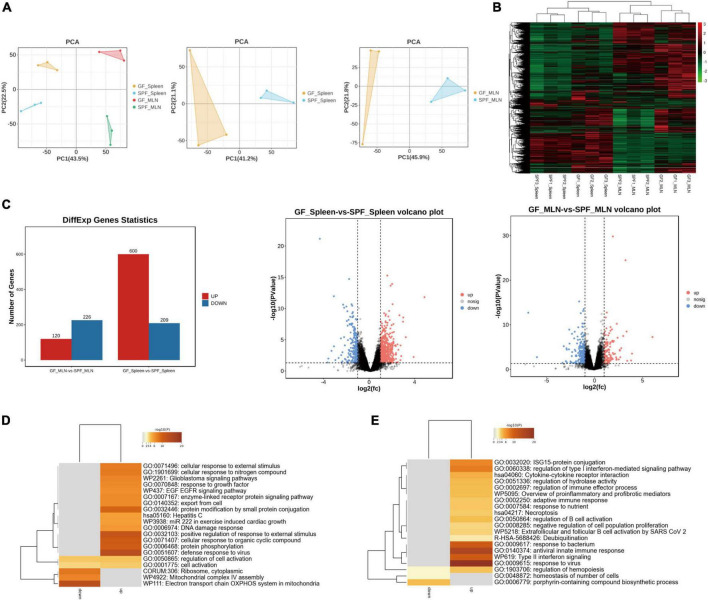
Expression profiles of mRNA in the spleen and MLNs in GF and SPF piglets. **(A)** PCA of the spleen and MLNs in the GF and SPF piglets. **(B)** Hierarchical clustering based on global mRNA expression profiles in the spleen or MLNs. **(C)** Numbers of upregulated and downregulated DE mRNAs derived from volcano plots. Functional enrichment analysis of DE mRNAs in the spleen **(D)** and MLNs **(E)**; *n* = 3.

RNA sequencing (RNA-seq) was used to explore the gene expression patterns of spleen and MLNs in the presence or absence of the commensal microbiota. We obtained ∼218.10 G clean data from 12 libraries (18.175 G per library) (Supplementary Table 2), with a total of 12,434 mRNAs identified. Principal component analysis (PCA) and hierarchical clustering showed different expression patterns between the spleens and MLNs of the GF and SPF piglets (Figures 3A, B). A total of 809 (600 upregulated and 209 downregulated) and 346 (120 upregulated and 226 downregulated) DE mRNAs were obtained in the spleen and MLNs, respectively (Figure 3C and Supplementary Table 3). We had obtained some highly upregulated genes, such as TNF receptor superfamily (TNFRSF) was found in spleen and MLNs upregulated genes, namely TNFRSF19 and TNFRSF17, which function to activate signaling pathways for development, organogenesis, cell death, and survival (Zhao et al., 2018). In addition, USP1 associated with innate immunity (Zhai et al., 2024), was also found in the upregulated genes, and IRF7 expressed in lymphocytes consistented with the above results (Li et al., 2021). Gene Ontology (GO) enrichment showed that many immune-related pathways, such as cellular response to external stimulus and defense response to virus were enriched in the spleen, while adaptive immune response and antiviral innate immune response were enriched in MLNs (Figures 3D, E and Supplementary Table 4). These results indicated that colonization of the commensal microbiota enhances the expression of immune-related signaling pathways and promotes the function of the immune system.

### 3.4 Effects of the commensal microbiota on innate and adaptive immune-related signaling pathways in the piglet spleen and MLNs

**FIGURE 4:**
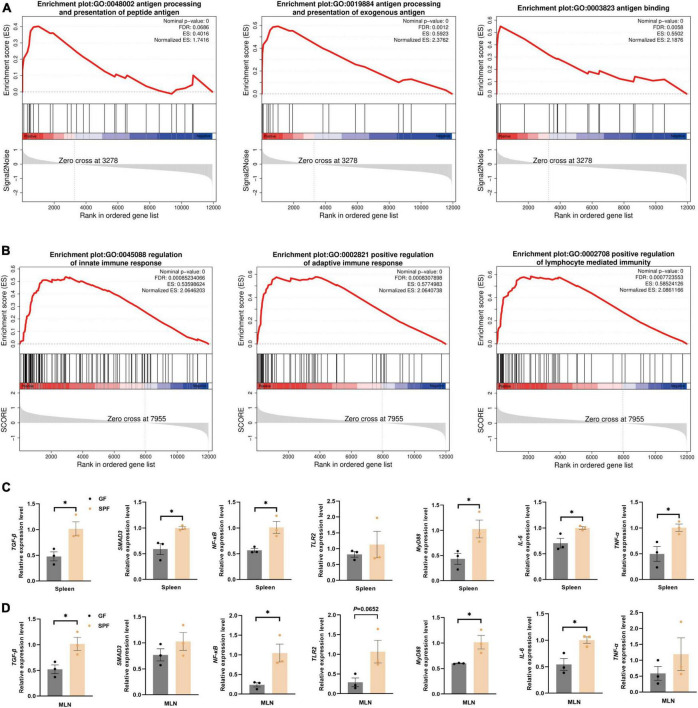
Significant activation of immune-related pathways in the spleen and MLNs. GSEA of gene sets involved in immune-related pathways in the spleen **(A)** and MLNs **(B)**. **(C,D)** RT-qPCR assays of relative expression of the core genes identified in panels **(A,B)** using *GAPDH* as the internal reference gene. *n* = 3. *P < 0.05.

The colonization of the commensal microbiota significantly stimulated the immune status of the spleen and MLNs. Therefore, we used the GO database to download genes and products related to inflammatory factors (Supplementary Table 5). In the spleen and MLNs, the majority of genes in the gene set of the SPF piglets was significantly upregulated (Supplementary Figure 2). However, gene set analysis only focused on specific related pathway. Therefore, we used GSEA to analyze the overall gene expression data of the spleen and MLNs, focusing on the synergistic changes of the entire gene set, and exploring the impact of the commensal microbiota on the overall immune-related signaling pathways in the spleen and MLNs. Compared with the GF piglet, the antigen processing and presentation of exogenous antigen-related signaling pathways (GO:0048002, GO:0019884, and GO:0003823) were significantly activated in the spleen of the SPF piglet (Figure 4A). The SPF piglet also showed not only activation of the innate and adaptive signaling pathways (GO:0045088 and GO:0002821), but also positive regulation of the lymphocyte-mediated immunity signaling pathway (G0:0002708) in MLNs compared with the GF piglet (Figure 4B). In addition, RT-qPCR was used to verify the vital factors regulating inflammatory signaling pathways and inflammation-related factors triggered by the antigen response shown in the results of GSEA. We detected the expression of an immune-suppressive cytokine (TGF-β)-related signaling pathway and found that the expression levels of *TGF-β* and *SMAD3* were higher in the spleen and MLNs of the SPF piglet compared with those in the GF piglet (Figures 4C, D). Additionally, the expression levels of *TLR2*, *NF-κB*, *MyD88*, and proinflammatory factor genes *IL-6* and *TNF-α* were also higher in the spleen and MLNs of the SPF piglet compared with those in the GF piglet (Figures 4C, D). These results revealed that the commensal microbiota impacts the specific immune-related signaling pathways of the spleen and MLNs, enhancing the expression of immune response pathways, including the TGF-β signaling pathway, and inflammatory signaling pathways (TLR2/MyD88/NF-κB).

### 3.5 Effects of the commensal microbiota on immune cellular abundance in the spleen and MLNs

**FIGURE 5:**
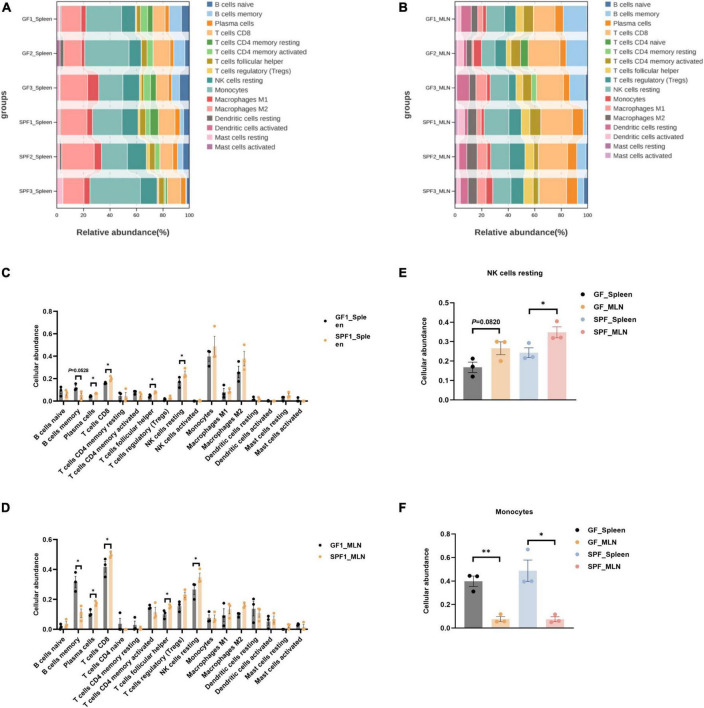
Deconvolution analysis of the spleen and MLNs using CIBERSORTx. Abundances of all identifiable immune cell types in the spleen **(A,C)** and MLNs **(B,D)**. Abundances of resting NK cells **(E)** and monocytes **(F)** in the spleen and MLNs. *n* = 3. *P < 0.05, **P < 0.01.

The commensal microbiota regulates the function and abundance of multiple immune cell types (Scott and Mann, 2020). CIBERSORTx is a suitable tool for the assessment of cellular abundance and cell type-specific gene expression patterns from bulk tissue transcriptome profiles (Steen et al., 2020). We identified 16 and 17 immune cell types in the spleen and MLNs, respectively, using CIBERSORTx (Figures 5A, B). The abundances of plasma cells, CD8^+^ T cells, follicular helper T cells, and resting NK cells were significantly increased in the spleen and MLNs of the SPF piglet compared with those in the GF piglet (*P* < 0.05; Figure 5C). By contrast, the abundance of memory B cells was lower in the spleen and MLNs of the SPF piglet than in the GF piglet (Figure 5D). In addition, compared with the spleen, the abundances of resting NK cells and monocytes in MLNs were higher and lower, respectively (Figures 5E, F). These results indicated that the commensal microbiota alters the abundance of immune cells by influencing the cell type-specific gene expression patterns of spleen and MLNs. In addition, the commensal microbiota had different effects on the abundances of resting NK cells and monocytes.

### 3.6 Immune-related pathways verified by splenic lymphocyte culture

**FIGURE 6:**
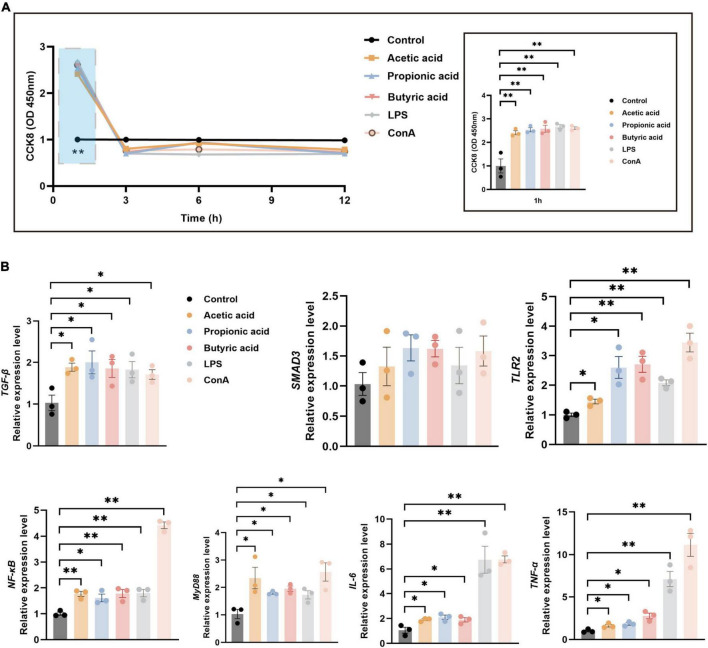
Splenic lymphocyte proliferation and mRNA expression under various treatments. **(A)** CCK-8 assays of splenic lymphocyte proliferation following treatment with acetic acid, propionic acid, and butyric acid, B cell irritant LPS, or T cell irritant ConA. The right panel shows a detailed view of the blue boxed area in the left panel. **(B)** RT-qPCR analysis of relative mRNA expression of immune-related pathway genes at 1 h post-treatment using *GAPDH* as the internal reference gene. *n* = 3. *P < 0.05, **P < 0.01.

To verify the immune-related pathways underlying the regulation of tissue immunity function, we established an *in vitro* model in splenic lymphocytes (Supplementary Figure 3). Cell proliferation was significantly increased under treatment with acetic acid, propionic acid, butyric acid, LPS, or ConA for 1 h compared with control (*P* < 0.01; Figure 6A). However, proliferation dropped thereafter, with no significant differences between the treatment and control groups 3, 6, and 12 h of treatment (Figure 6A). Next, we used RT-qPCR to analyze the expression of immune-related pathway genes at 1 h post-treatment, including TGF-β/SMAD3 and TLR2/MyD88/NF-κB pathway genes, as well as inflammatory factor genes *IL-6* and *TNF-α*. The expression levels of *TGF-β* were significantly increased under all treatments compared with control (*P* < 0.05; Figure 6B), while the expression of *SMAD3* showed an increasing trend under all treatments (Figure 6B). The expression levels of *TLR2*, *MyD88*, *NF-κB*, *IL-6*, and *TNF-*α were also significantly increased in all treatment groups compared with control (Figure 6B). These results revealed that the tendency for expression of specific immune-signal pathways in the spleen and MLNs *in vivo* were consistent with those in lymphocytes *in vitro*.

## 4 Discussion

Removal of the commensal microbiota leads to defects in the immune system and incomplete immune cell development (Antonini et al., 2019). At present, the impacts of the commensal microbiota on the immune system are mostly being studied in GF mice or pseudo-GF mice treated with antibiotics (Ahmed et al., 2021). Nevertheless, the porcine immune system has several peculiarities that separate it from that of mice. For instance, pigs have a high frequency of peripheral CD4^+^ and CD8^+^ effector/memory T cells (Gu et al., 2022). Furthermore, compared with mice, pigs are more anatomically and immunologically similar to humans (Wang and Donovan, 2015). GF and SPF piglets provide ideal animal models for investigating the impacts of the commensal microbiota on the immune function of the spleen and MLNs.

The spleen in piglets with colonization of the commensal microbiota exhibited significantly higher weight and organ index in this study. The same results were also confirmed in the spleen and MLNs of antibiotic-treated mice (Durand et al., 2018; Thackray et al., 2018). Furthermore, the presence of the commensal microbiota promoted the development of red pulp and germinal centers in the spleen and MLNs, respectively. In the absence of the commensal microbiota, the same immune structure was present, but was underdeveloped. This finding suggested that the commensal microbiota, instead of impacting organogenesis, influenced immune system development (Gensollen et al., 2016). Furthermore, the commensal microbiota increased the distribution areas of specific B cells and T cells in the spleen. These results were confirmed in piglet PPs (Zhang et al., 2023), indicating that the commensal microbiota is crucial for the development of immune tissues and the differentiation and maturation of B and T cells (Wang et al., 2019).

Many immune-related DE mRNAs in the spleen and MLNs between GF and SPF piglets were observed in this study, which is consistent with previous findings in mice (Wang et al., 2023b). Conventional pigs and GF pigs also exhibit many immune-related DE mRNAs in the spleen (Sun et al., 2018). Interestingly, our functional enrichment analysis showed that spleen-specific and MLNs-specific DE mRNAs were involved in response to virus and antiviral innate immune response. These findings are consistent with previous studies showing that the commensal microbiota influences resistance to external stimuli aspects of the jejunum, colon, spleen, and oral mucosa (Sun et al., 2018). Additionally, our team confirmed the importance of the commensal microbiota in the phenotypic characteristics and gene expression of PPs in piglets (Zhang et al., 2023).

We used the GO database of immunomarker genes as a reference for the GSEA to identify specific signaling pathways that influence the immune-related functions of the spleen and MLNs. This revealed that antigen processing and presentation of exogenous, innate immunity and adaptive immunity had far greater enrichment in the spleen and MLNs under colonization of the commensal microbiota. Compared with GF animals, the presence of the commensal microbiota significantly stimulated the host immune system, thereby achieving its purpose of self-protection (Jin et al., 2019; Gola et al., 2021). Inflammatory signaling pathway expression and activation of inflammatory factors indicate that homeostasis is maintained through an active immunoregulatory process (Huang et al., 2005). In the presence of the commensal microbiota, RT-qPCR showed increased expression of the TGF-β/SMAD3 signaling pathway in this study. The dominant function of TGF-β is to regulate peripheral immune homeostasis, particularly in the microbe-rich, antigen-rich environment of the gut. The SMAD pathway regulates the production of IgA by B cells, maintains the protective mucosal barrier, and promotes the balanced differentiation of CD4^+^ T cells into inflammatory T helper 17 cells and suppressive *FOXP3*-expressing Tregs (Malhotra and Kang, 2013). Under treatment with SCFAs, LPS, and ConA, the expression levels of *TLR2*, *MyD88*, and *NF-κB* increased in the spleen and MLNs of the SPF piglet, as well as in splenic lymphocytes. Resident enteric bacteria activated intestinal *IL-10*-producing B cells through the TLR2/MyD88/PI3K pathway to reduce colonic T cell activation and maintain mucosal homeostasis (Mishima et al., 2019). Consistently, TLRs significantly enhance the ability of DCs to present antigen through the TLR/MyD88/NF-κB signaling pathway (Hu et al., 2016). The release of proinflammatory cytokines is one way that the body resists infection of tissues or cells by pathogenic bacteria. An appropriate amount of proinflammatory cytokines regulates the immune response to some extent, leading to resistance or clearing of pathogens, promoting the repair of damaged tissues, inducing tumor cell apoptosis, and so forth (Rostami et al., 2013). Consistent with other studies, we confirmed the impact of SCFAs on the metabolism of immune cells, including immunosuppressive and inflammatory cells (Wang et al., 2023a). These data in piglets support the notion that the colonization of the commensal microbiota increases the expression of proinflammatory factors that, together with timely expression of inflammatory suppressive factors that inhibit their overexpression, maintain the immune homeostasis of the body.

The effect of the commensal microbiota on the abundance of immune cells has been investigated in multiple organs in mice and humans (Papotto et al., 2021). CIBERSORTx is an emerging technology that identifies cell types based on relative subsets of known RNA transcripts without physical cell sorting (Wang et al., 2021), and has been validated in multiple tumor types (He et al., 2023). Using CIBERSORTx, we were surprised to find that the commensal microbiota significantly affected the abundances of innate and adaptive immune cells in the spleen and MLNs, especially those of memory B cells, plasma cells, multiple T cell types, and resting NK cells. It also revealed fewer B cells in the ileum and blood in the presence of the commensal microbiota or viruses in SPF piglets compared with those in GF piglets (Sinkora et al., 2014; Potockova et al., 2015). Stimulation of commensal microbes also led to significantly greater abundance of plasma cells in the SPF piglet than in the GF piglet, consistent with the decrease in IgA-producing plasma cells in GF mice (Liu and Rhoads, 2013). Additionally, commensal microbes promoted the abundances of related T cell types, consistent with previously reported results (Ivanov et al., 2022). Moreover, colonization of the commensal microbiota led to the appearance of effector CD4^+^CD8^+^ αβ T helper and CD2^+^CD8^–^ γδ T cells (Sinkora et al., 2011), while colonization with *Staphylococcus aureus* was negatively associated with the abundance of memory B cells (Kandasamy et al., 2014). Although commensal microbes are known to affect the abundance of lymphocytes (Sinkora and Butler, 2016), the reasons for the significant differences in memory B cells require further exploration. Direct contact between MLNs and the commensal microbiota resulted in significantly greater abundance of NKs in the MLNs compared to that in the spleen of the SPF piglet. The abundance of NK cells in different ecological niches of the small intestine also varied (Potockova et al., 2015). Similarly, the abundance of monocytes, as peripheral immune cells, was significantly greater in the spleen than in the MLNs. These findings suggested that colonization of the commensal microbiota had varying degrees of impact on immune cells in different parts of the piglet body, as previously shown in mice (Van Praet et al., 2015).

CIBERSORTx algorithm was accurate and feasible to analyze the proportion of immune cells in tissues using RNA-seq data, which has been repeatedly mentioned in many journals (Newman et al., 2019; Steen et al., 2020; Wang et al., 2021). However, the use of bulk RNA-seq data for deconvolution analysis in this study had the following shortcomings. First, because the CIBERSORTx database features only 22 specific types of immune cells, we were unable to obtain the exact types and abundance of all immune cells. Second, the resolution of bulk RNA-seq was insufficient in that it only captures the average expression level of immune tissues. Third, spleen and MLNs exhibit tissue heterogeneities in terms of cell subpopulations and functions associated with physiological processes that RNA-seq cannot fully elucidate. In future studies, we aim to explore this issue further by combining single-cell RNA-seq and spatial transcriptome technologies to explore the effects of the commensal microbiota on the precise cellular composition of distinct tissue regions and spatial gene expression of the immune system.

## 5 Conclusion

The presence of the commensal microbiota promoted the development and maturation of the spleen and MLNs, impacting the abundance of various immune cell types and the proliferation and differentiation of lymphocytes. This work also explored microbial–host interactions and provided evidence to support the use of the piglet immunological model as an alternative to widely used rodent models.

## Data availability statement

The original contributions presented in the study are publicly available. The RNA-seq data have been deposited in the Genome Sequence Archive (GSA) with the accession number CRA015219.

## Ethics statement

The animal studies were approved by the Chongqing Academy of Animal Science. The studies were conducted in accordance with the local legislation and institutional requirements. Written informed consent was obtained from the owners for the participation of their animals in this study.

## Author contributions

YL: Writing – original draft, Writing – review & editing. JZ: Writing – original draft, Writing – review & editing. GY: Validation, Writing – review & editing. CT: Data curation, Writing – review & editing. XKL: Data curation, Writing – review & editing. LL: Formal analysis, Writing – review & editing. KL: Formal analysis, Writing – review & editing. JS: Validation, Writing – review & editing. YD: Validation, Writing – review & editing. XWL: Formal analysis, Writing – review & editing. ML: Formal analysis, Writing – review & editing. LG: Supervision, Writing – review & editing. JM: Supervision, Writing – review & editing.

## Footnotes

1
https://metascape.org/gp/index.html#/main/step1

2
https://www.omicshare.com/

3
https://cibersortx.stanford.edu/
