# Supplementary material for: Effects of the commensal microbiota on spleen and mesenteric lymph node immune function: investigation in a germ-free piglet model
Source: Front Microbiol. 2024 Jun 12;15:1398631. doi: 10.3389/fmicb.2024.1398631 (PMC11201156; doi:10.3389/fmicb.2024.1398631)
Supplement: Supplementary file 6 [file Data_Sheet_1.PDF]

# *Supplementary Material*

## Supplementary Figures

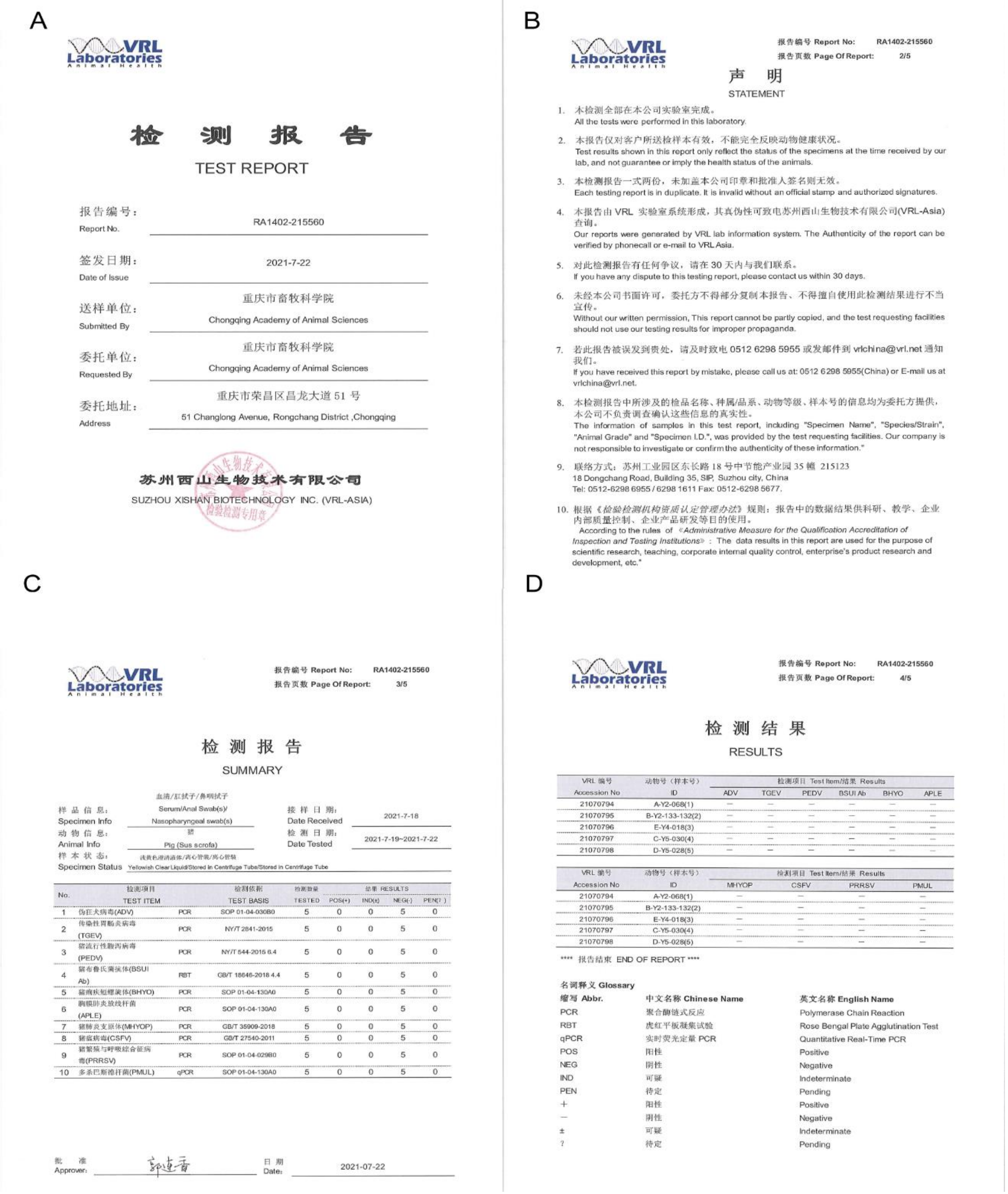

A

VRL Laboratories Animal Health

检 测 报 告

TEST REPORT

| | |
|---|---|
| 报告编号：Report No. | RA1402-215560 |
| 签发日期：Date of Issue | 2021-7-22 |
| 送样单位：Submitted By | 重庆市畜牧科学院 Chongqing Academy of Animal Sciences |
| 委托单位：Requested By | 重庆市畜牧科学院 Chongqing Academy of Animal Sciences |
| 委托地址：Address | 重庆市荣昌区昌龙大道 51 号 51 Changlong Avenue, Rongchang District ,Chongqing |

苏州西山生物技术有限公司

SUZHOU XISHAN BIOTECHNOLOGY INC. (VRL-ASIA)

B

VRL Laboratories Animal Health

报告编号 Report No: RA1402-215560

报告页数 Page Of Report: 2/5

声 明

STATEMENT

1. 本检测全部在本公司实验室完成。
All the tests were performed in this laboratory.
2. 本报告仅对客户所送检样本有效，不能完全反映动物健康状况。
Test results shown in this report only reflect the status of the specimens at the time received by our lab, and not guarantee or imply the health status of the animals.
3. 本检测报告一式两份，未加盖本公司印章和批准人签名则无效。
Each testing report is in duplicate. It is invalid without an official stamp and authorized signatures.
4. 本报告由 VRL 实验室系统形成，其真伪性可致电苏州西山生物技术有限公司(VRL-Asia)查询。
Our reports were generated by VRL lab information system. The Authenticity of the report can be verified by phonecall or e-mail to VRL Asia.
5. 对此检测报告有任何争议，请在 30 天内与我们联系。
If you have any dispute to this testing report, please contact us within 30 days.
6. 未经本公司书面许可，委托方不得部分复制本报告、不得擅自使用此检测结果进行不当宣传。
Without our written permission, This report cannot be partly copied, and the test requesting facilities should not use our testing results for improper propaganda.
7. 若此报告被误发到贵处，请及时致电 0512 6298 5955 或发邮件到 vrlchina@vrl.net 通知我们。
If you have received this report by mistake, please call us at: 0512 6298 5955(China) or E-mail us at vrlchina@vrl.net.
8. 本检测报告中所涉及的检品名称、种属/品系、动物等级、样本号的信息均为委托方提供，本公司不负责调查确认这些信息的真实性。
The information of samples in this test report, including "Specimen Name", "Species/Strain", "Animal Grade" and "Specimen I.D.", was provided by the test requesting facilities. Our company is not responsible to investigate or confirm the authenticity of these information."
9. 联络方式：苏州工业园区东长路 18 号中节能产业园 35 幢 215123
18 Dongchang Road, Building 35, SIP, Suzhou city, China
Tel: 0512-6298 6955 / 6298 1611 Fax: 0512-6298 5677.
10. 根据《*检验检测机构资质认定管理办法*》规则：报告中的数据结果供科研、教学、企业内部质量控制、企业产品研发等目的使用。
According to the rules of 《*Administrative Measure for the Qualification Accreditation of Inspection and Testing Institutions*》: The data results in this report are used for the purpose of scientific research, teaching, corporate internal quality control, enterprise's product research and development, etc."

C

VRL Laboratories Animal Health

报告编号 Report No: RA1402-215560

报告页数 Page Of Report: 3/5

检 测 报 告

SUMMARY

| | | | |
|---|---|---|---|
| 样品信息：Specimen Info | 血清/肛拭子/鼻咽拭子 Serum/Anal Swab(s)/ Nasopharyngeal swab(s) | 接样日期：Date Received | 2021-7-18 |
| 动物信息：Animal Info | 猪 Pig (Sus scrofa) | 检测日期：Date Tested | 2021-7-19~2021-7-22 |
| 样本状态：Specimen Status | 浅黄色澄清液体/离心管装/离心管装 Yellowish Clear Liquid/Stored in Centrifuge Tube/Stored in Centrifuge Tube | | |

| No. | 检测项目 TEST ITEM | | 检测依据 TEST BASIS | 检测数量 TESTED | 结果 RESULTS POS(+) | IND(±) | NEG(-) | PEN(?) |
|---|---|---|---|---|---|---|---|---|
| 1 | 伪狂犬病毒(ADV) | PCR | SOP 01-04-030B0 | 5 | 0 | 0 | 5 | 0 |
| 2 | 传染性胃肠炎病毒 (TGEV) | PCR | NY/T 2841-2015 | 5 | 0 | 0 | 5 | 0 |
| 3 | 猪流行性腹泻病毒 (PEDV) | PCR | NY/T 544-2015 6.4 | 5 | 0 | 0 | 5 | 0 |
| 4 | 猪布鲁氏菌抗体(BSUI Ab) | RBT | GB/T 18646-2018 4.4 | 5 | 0 | 0 | 5 | 0 |
| 5 | 猪痢疾短螺旋体(BHYO) | PCR | SOP 01-04-130A0 | 5 | 0 | 0 | 5 | 0 |
| 6 | 胸膜肺炎放线杆菌 (APLE) | PCR | SOP 01-04-130A0 | 5 | 0 | 0 | 5 | 0 |
| 7 | 猪肺炎支原体(MHYOP) | PCR | GB/T 35909-2018 | 5 | 0 | 0 | 5 | 0 |
| 8 | 猪瘟病毒(CSFV) | PCR | GB/T 27540-2011 | 5 | 0 | 0 | 5 | 0 |
| 9 | 猪繁殖与呼吸综合征病毒(PRRSV) | PCR | SOP 01-04-029B0 | 5 | 0 | 0 | 5 | 0 |
| 10 | 多杀巴斯德杆菌(PMUL) | qPCR | SOP 01-04-130A0 | 5 | 0 | 0 | 5 | 0 |

批准 Approver: 郭遠香　　日期 Date: 2021-07-22

D

VRL Laboratories Animal Health

报告编号 Report No: RA1402-215560

报告页数 Page Of Report: 4/5

检 测 结 果

RESULTS

| VRL 编号 Accession No | 动物号（样本号）ID | 检测项目 Test Item/结果 Results ADV | TGEV | PEDV | BSUI Ab | BHYO | APLE |
|---|---|---|---|---|---|---|---|
| 21070794 | A-Y2-068(1) | — | — | — | — | — | — |
| 21070795 | B-Y2-133-132(2) | — | — | — | — | — | — |
| 21070796 | E-Y4-018(3) | — | — | — | — | — | — |
| 21070797 | C-Y5-030(4) | — | — | — | — | — | — |
| 21070798 | D-Y5-028(5) | — | — | — | — | — | — |

| VRL 编号 Accession No | 动物号（样本号）ID | 检测项目 Test Item/结果 Results MHYOP | CSFV | PRRSV | PMUL |
|---|---|---|---|---|---|
| 21070794 | A-Y2-068(1) | — | — | — | — |
| 21070795 | B-Y2-133-132(2) | — | — | — | — |
| 21070796 | E-Y4-018(3) | — | — | — | — |
| 21070797 | C-Y5-030(4) | — | — | — | — |
| 21070798 | D-Y5-028(5) | — | — | — | — |

**** 报告结束 END OF REPORT ****

名词释义 Glossary

| 缩写 Abbr. | 中文名称 Chinese Name | 英文名称 English Name |
|---|---|---|
| PCR | 聚合酶链式反应 | Polymerase Chain Reaction |
| RBT | 虎红平板凝集试验 | Rose Bengal Plate Agglutination Test |
| qPCR | 实时荧光定量 PCR | Quantitative Real-Time PCR |
| POS | 阳性 | Positive |
| NEG | 阴性 | Negative |
| IND | 可疑 | Indeterminate |
| PEN | 待定 | Pending |
| + | 阳性 | Positive |
| — | 阴性 | Negative |
| ± | 可疑 | Indeterminate |
| ? | 待定 | Pending |

**Supplementary Figure S1.** SPF piglet pathogen detection. (A) Test report number and time, (B)Testing statement, (C)Summary, (D)Results.

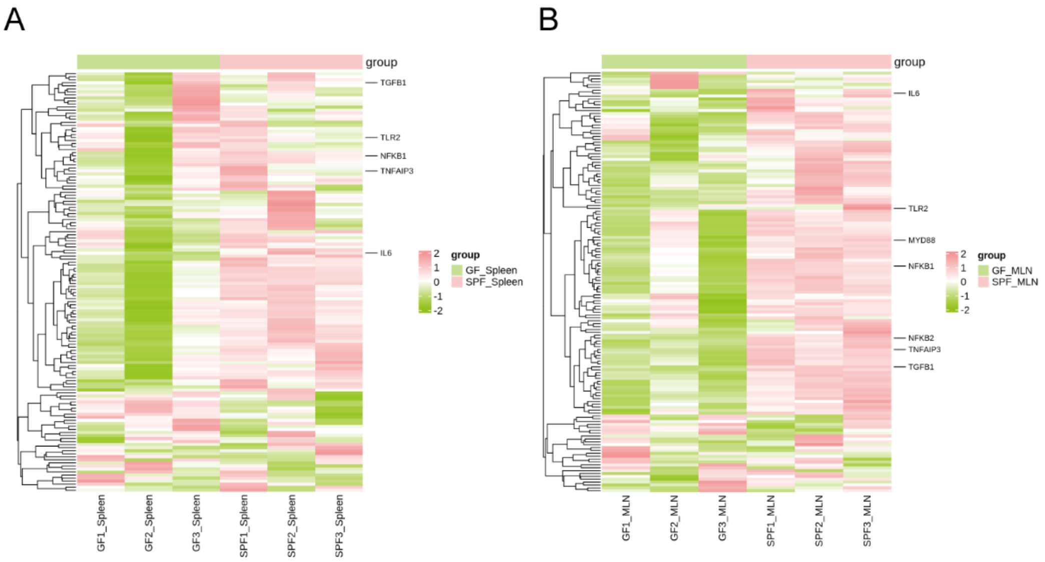


**Supplementary Figure S2.** Gene set analysis based on RNA-seq. (A, B) Gene set analysis related to the spleen (A) and MLNs (B) inflammatory factors.

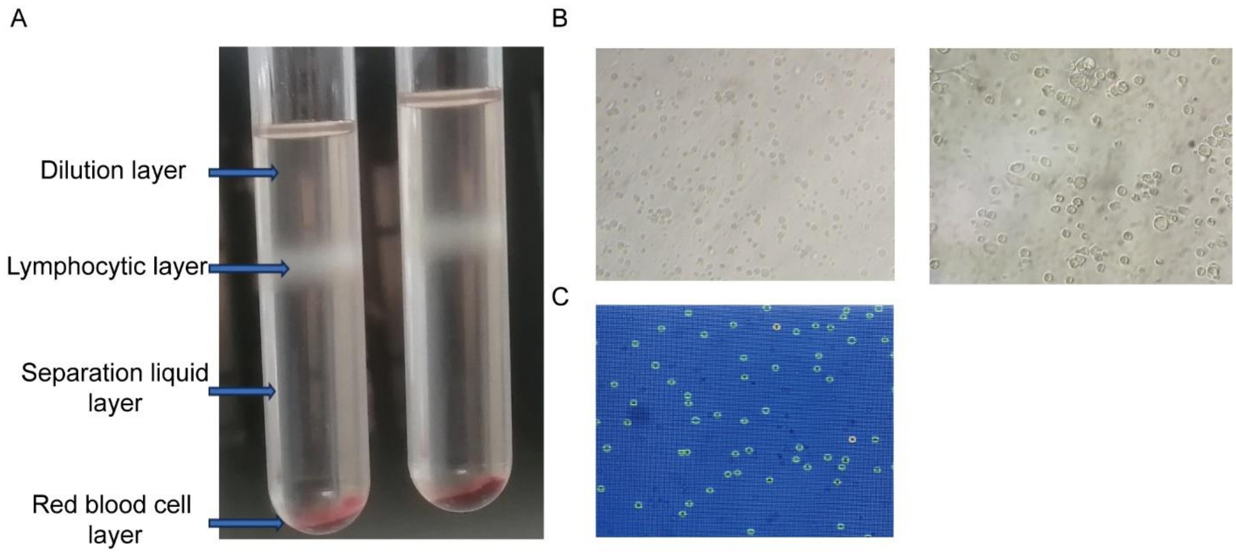


**Supplementary Figure S3.** Isolation of piglet splenic lymphocytes. (A) Splenic lymphocytes stratification, (B) Splenic lymphocytes under inverted microscope, 10 × (left), 20× (right), (C) Results of spleen lymphocyte trypan blue count, 10 ×.
